# MBG: Minimizer-based sparse de Bruijn Graph construction

**DOI:** 10.1093/bioinformatics/btab004

**Published:** 2021-01-21

**Authors:** Mikko Rautiainen, Tobias Marschall

**Affiliations:** Center for Bioinformatics, Saarland University, 66123 Saarbrücken, Germany; Max Planck Institute for Informatics, 66123 Saarbrücken, Germany; Saarbrücken Graduate School for Computer Science, 66123 Saarbrücken, Germany; Heinrich Heine University Düsseldorf, Medical Faculty, Institute for Medical Biometry and Bioinformatics, 40225 Düsseldorf, Germany

## Abstract

**Motivation:**

De Bruijn graphs can be constructed from short reads efficiently and have been used for many purposes. Traditionally, long-read sequencing technologies have had too high error rates for de Bruijn graph-based methods. Recently, HiFi reads have provided a combination of long-read length and low error rate, which enables de Bruijn graphs to be used with HiFi reads.

**Results:**

We have implemented MBG, a tool for building sparse de Bruijn graphs from HiFi reads. MBG outperforms existing tools for building dense de Bruijn graphs and can build a graph of 50× coverage whole human genome HiFi reads in four hours on a single core. MBG also assembles the bacterial *E.coli* genome into a single contig in 8 s.

**Availability and implementation:**

Package manager: https://anaconda.org/bioconda/mbg and source code: https://github.com/maickrau/MBG.

**Supplementary information:**

Supplementary data are available at *Bioinformatics* online.

## 1 Introduction

De Bruijn graphs (DBGs) have been used in sequence analysis for purposes such as genome assembly (Bankevich *et al.*, 2012; Garg *et al.*, 2018; Pevzner et al., 2001; Wick et al., 2017) and error correction (Miclotte et al., 2016; Rautiainen and Marschall, 2019; Salmela and Rivals, 2014). Sparse de Bruijn graphs (Ye et al., 2011) are a form of DBGs which use only a subset of k-mers and so reduce runtime and memory use. Minimizer winnowing (Roberts et al., 2004; Schleimer et al., 2003) is a method of selecting a subset of k-mers from a sequence, which has been applied to building sparse DBGs (Coombe *et al.*, 2020). Recently, HiFi reads (Wenger et al., 2019) have reached read lengths of thousands of base pairs with error rates comparable or superior to short reads. The combination of long-read lengths and low error rates makes DBGs an attractive idea for HiFi reads and might enable hybrid methods, which typically use a combination of Illumina and long reads, to use a combination of HiFi and even longer ONT reads (Logsdon *et al.*, 2020). Increasing the k-mer size leads to better repeat resolution and therefore better assembly. However, current tools do not scale to k-mer sizes in thousands.

*Contributions.* We have implemented the tool MBG (**M**inimizer-based sparse de **B**ruijn **G**raph) for constructing sparse de Bruijn graphs. MBG selects k-mers by minimizer winnowing (Schleimer *et al.*, 2003) and builds the graph from those k-mers. This approach has previously been used in the ntJoin scaffolder (Coombe *et al.*, 2020) for building graphs from assembled contigs to scaffold assemblies.

MBG can construct graphs with arbitrarily high k-mer sizes, and we show that k-mer sizes of thousands of base pairs are practical with real HiFi read data. MBG outperforms existing de Bruijn graph construction tools in runtime, with a runtime of only a few hours on a single core for constructing a graph of 50× coverage whole human genome HiFi reads.

## 2 Materials and methods

We give a brief overview of the implementation here with detailed explanations of the individual steps in Supplementary Note SA. Since most errors in HiFi reads are homopolymer run length errors (Wenger *et al.*, 2019), the input reads are homopolymer compressed by collapsing homopolymer runs into one character. Homopolymer compression removes most errors but it might also lead to repeat collapses if there are long repeats which only differ in homopolymer run lengths. A rolling hash function (Mohamadi *et al.*, 2016) is used to assign a hash value to each k-mer. Minimizer winnowing (Schleimer *et al.*, 2003) is then used to select a subset of k-mers. The selected k-mers are compressed by hashing them into 128-bit integers, which form the nodes of the minimizer graph. Edges are added whenever two minimizers are adjacent to each other in the reads. Transitive edges caused by sequencing errors are cleaned. Non-branching paths of the graph are then condensed into unitigs. Finally, the 128-bit hashes are replaced with their base pair sequences, and homopolymer runs are expanded. The graph is then written in the GFA format (Li, 2016).

## 3 Results

We built sparse de Bruijn graphs using HiFi read data. We varied the k-mer size *k*, and for MBG, the window size parameter *w* (Schleimer *et al.*, 2003), which determines the sparseness of the resulting graph, with higher *w* leading to sparser graphs. Details of the experimental setup are in Supplementary Note SB. Table 1 shows the results for selected parameters and Supplementary Table S1 contains the full results.

**Table 1.** Experimental results

Dataset	Tool	k	w	CPU-time	Memory (Gb)	N50
*E.coli*	BCalm2	61	–	0:00:59	1.1	1 025
		127	–	0:01:40	2.0	1 212
		501	–	0:17:11	3.6	4 999
		1001	–	1:42:26	3.5	13 688
		2001	–	8:12:45	3.8	5 908
		3001	–	10:33:11	4.0	4 393
*E.coli*	MBG	61	1	0:01:33	3.2	73 728
		61	10	0:00:25	0.6	82 427
		61	20	0:00:16	0.3	82 418
		61	30	0:00:13	0.3	82 394
		127	1	0:01:53	3.9	132 765
		127	10	0:00:30	0.8	132 569
		127	20	0:00:17	0.4	132 766
		127	30	0:00:13	0.3	132 764
		501	500	0:00:10	0.12	177 653
		1001	1000	0:00:09	0.13	698 111
		2001	2000	0:00:08	0.14	4 639 237
		2501	2500	0:00:08	0.14	4 644 046
		3001	3000	0:00:08	0.14	4 090 727
HG002	BCalm2	127	–	32:00:32	6.4	249
HG002	MBG	2001	2000	4:07:22	138.5	12 095
		3001	3000	3:57:59	137.3	23 104
		4001	4000	3:35:03	120.4	26 736
		5001	5000	2:06:52	68.9	20 699
		2001	400	6:01:18	250.0	8 669
		3001	600	6:05:17	243.9	15 085
		4001	800	3:55:50	245.3	23 868
		5001	1000	4:47:44	244.1	33 649

*Comparison to existing tools*. We compared MBG to BCalm2 (Chikhi *et al.*, 2016) for building graphs using HiFi reads of *E.coli*. Note that N50 is not directly comparable between MBG and BCalm2 since the homopolymer compression step removes most errors and therefore greatly improves N50. BCalm2 uses less memory than MBG with *w *=* *1, but for *w *=* *10 and higher MBG uses less memory. MBG is faster than BCalm2 when *w *>* *1, and slightly slower with *w *=* *1. The runtime of BCalm2 increases greatly as the k-mer size increases while MBG scales efficiently to high *k*. Due to homopolymer errors in the reads, the N50 for BCalm2 suffers when *k* grows above 1001. On the other hand, the homopolymer compression of MBG enables it to scale to higher *k*. With higher *w* MBG is an order of magnitude faster than BCalm2. Supplementary Table S2 and Supplementary Note SB contain an evaluation of the error rates of the assemblies. With *k *=* *2501 and *w *=* *2500, MBG assembles *E. coli* correctly into a single contig in 8 s on a single core with an estimated error rate of 45 errors per 100kbp. Almost all errors are homopolymer run length errors, with only 0.18 non-homopolymer errors per 100kbp.

*Whole human genome HiFi*. We ran MBG on whole human genome HiFi data from the individual HG002. Runtime is between 2 and 7 h on a single core with all parameter sets, showing that MBG is fast and scales to large *k*. The limitation on increasing *k* and *w* even higher is the error rate and read length of the HiFi reads. We also ran BCalm2 on the same reads with *k *=* *127. We did not run BCalm2 with higher *k* since the previous experiment suggests the runtime would be prohibitive. MBG is an order of magnitude faster than BCalm2, however, memory use is higher since MBG keeps all data in memory while BCalm2 uses temporary files on disk.

## 4 Conclusion

We have implemented MBG, a tool for building sparse de Bruijn graphs from HiFi reads using minimizer winnowing. The sparsification enables MBG to run orders of magnitude faster than tools for building dense de Bruijn graphs. Increasing the sparsity parameter *w* speeds up assembly but can reduce homopolymer run length consensus accuracy. MBG uses a novel method to compress long k-mers to constant sized hashes and enables *k* to scale arbitrarily high.

MBG can quickly build de Bruijn graphs of mammalian sized genomes, with runtimes ranging from 2 to 7 h on a single core. The memory use currently prevents MBG from being ran on mammalian datasets on laptops and desktop computers. However, MBG fits comfortably in the RAM of most computing servers. Disk-based approaches used by previous tools (Chikhi *et al.*, 2016) might enable MBG to run on mammalian datasets on laptops. MBG enables small genomes such as *E. coli* to be assembled in a few seconds and mammalian genomes in a few hours.

*Financial Support*: none declared.

*Conflict of Interest*: none declared. 

## Data availability

Data used for the *E.coli* experiment is available in the Sequence Read Archive run SRR10971019. Data used for the HG002 experiment is available in Wenger *et al*. (2019). 

## Supplementary Material

btab004_Supplementary_Data
