# Supplementary material for: MBG: Minimizer-based sparse de Bruijn Graph construction
Source: Bioinformatics. 2021 Jan 21;37(16):2476–8. doi: 10.1093/bioinformatics/btab004 (PMC8521641; doi:10.1093/bioinformatics/btab004)
Supplement: btab004_Supplementary_Data [file btab004_supplementary_data.pdf]

Supplementary Material: MBG: Minimizer-based Sparse de Bruijn Graph Construction

A. Methods

Homopolymer compression. Most errors in HiFi reads are homopolymer run length errors (Wenger *et al.*, 2019). The sequences are first homopolymer compressed by collapsing homopolymer runs into one character, reducing the error rate by an order of magnitude. The lengths of the homopolymer runs are stored so that the original sequence can be reconstructed at the end.

Minimizer winnowing. MBG uses the rolling hash function from ntHash (Mohamadi *et al.*, 2016) to assign hash values to each k-mer of the input reads. The runtime of the rolling hash function is independent of k-mer size. In practice minimizer winnowing is the performance bottleneck of MBG, so we chose the ntHash method since it is the fastest hash we are aware of.

Minimizer winnowing (Schleimer *et al.*, 2003) is then applied to the k-mers given their hash values. The smallest k-mer in each window is selected for later processing. Selected k-mers which appear in the input data fewer times than a user given k-mer abundance cutoff are also discarded. Since the density of random minimizers is $\frac{2}{w}$ (Schleimer *et al.*, 2003), a window size of w will on average lead to a $\frac{w}{2}$-fold sparsity of selected k-mers.

Compressing arbitrary sized k-mers by hashing. The selected k-mers are compressed by hashing them into 128-bit integers. The 128-bit hashes are then used as the nodes of the graph. We used the c++ standard library's string hash function for building the hash. For a string s, the lower 64 bits of the hash are taken from the standard library hash of $s[1..\left\lfloor\frac{|s|}{2}\right\rfloor]$ and the upper 64 bits from the hash of $s[\left\lfloor\frac{|s|}{2}\right\rfloor+1..|s|]$. Note that hash quality is very important in this step. Since a hash collision would lead to two different sequences being represented by the same node, every k-mer must result in a unique hash to ensure correctness of the resulting graph.

Hash collisions. Given 128-bit random hashes, it is reasonable to assume that there are no hash collisions. To estimate the probability of a hash collision, the birthday paradox can be used. Given n k-mers to hash, and the size of the hash space $d = 2^{128}$, the probability of collision can be approximated with $p \approx 1 - e^{\frac{-n^2}{2d}}$. As of 1st July 2020, the size of the SRA database is 42441459655506377 base pairs. If the entire database were concatenated to one string and all of its k-mers for one k were hashed, there would be less than $4.3 * 10^{16}$ k-mers to hash. Applying the approximation of the birthday paradox to this number of k-mers gives a hash collision probability of $p \approx 1 - e^{\frac{-n^2}{2d}} < 1 - e^{\frac{-1.9*10^{33}}{2*2^{128}}} < 1 - e^{-10^{-5}} < 10^{-4}$ for hashing the entire SRA database. The probability of hash collision for any realistic dataset is therefore negligible assuming a random hash function. In addition, MBG checks for hash collisions during runtime. We have not seen a hash collision so far.

Transitive edge cleaning. Because the minimizers are sampled from a window, a sequencing error outside of a k-mer can affect whether the k-mer was chosen. That is, an error within a window but outside of the chosen k-mer in the error-free window can cause a different k-mer to be chosen in the error-containing window.

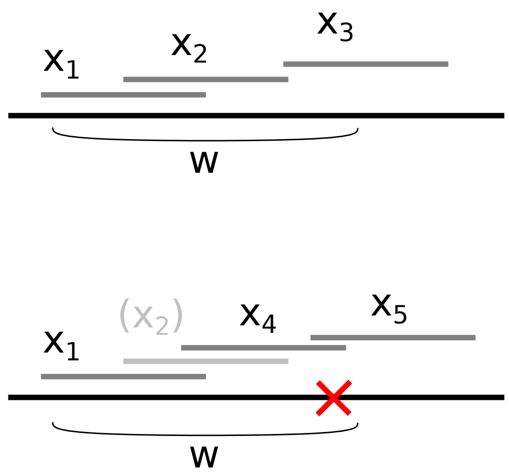

Figure 1: An illustration of the transitive edge problem. The top sequence (solid black line) has no errors and three k-mers, x_1, x_2 and x_3, are selected from it. The area marked by w is one window, from which x_2 was selected in the error-free sequence. The bottom sequence (solid black line) has a sequencing error (red cross). Due to the sequencing error, k-mer x_4 is selected from window w instead of x_2. The k-mers selected from the bottom sequence are x_1, x_4 and x_5. Even though the bottom sequence contains x_2 without errors, x_2 is not selected.

Figure 1 illustrates the problem. The error-free sequence has chosen k-mers x_1, x_2,

and x_3. The second sequence has a sequencing error outside of x_2 but covered by each window that selected x_2, causing x_2 to not be selected, and instead minimizers x_4 and x_5 are selected. If the minimizers were used as-is, the graph would have an extra edge $x_1 \to x_4$, and the correct edge $x_2 \to x_4$ would be missing.

To solve this, we look at all edges connecting minimizers. We build an *edge sequence* of the two adjacent minimizers. Given k-mers m_1 with sequence σ_1 and m_2 with sequence σ_2, and an overlap of b base pairs between them, the edge sequence is defined as $s = \sigma_1 + \sigma_2[b..k]$, that is, the concatenation of the two k-mers, taking into account not to duplicate the shared sequence in the overlap. Then, we check all k-mers in the edge sequence. If a k-mer m_3 inside the edge sequence was selected as a minimizer during minimizer winnowing, we mark the edge (m_1, m_2) as transitive, and add the edges (m_1, m_3) and (m_3, m_2) if they were not already present. Finally, we remove all transitive edges and transfer their read coverage to the replacement edges. In the example in Figure 1, this would remove the edge $x_1 \to x_4$, add the new edge $x_2 \to x_4$, and add the coverage of the removed edge $x_1 \to x_4$ to the edges $x_1 \to x_2$ and $x_2 \to x_4$.

Checking if a k-mer was selected as a minimizer takes $O(k)$ time and checking all k-mers in all edges would then take $O(mk^2)$ time for m minimizers. We improve the speed in practice by first using the rolling hash from minimizer winnowing to limit which k-mers to check. The hash values of the selected k-mers are stored, and then a k-mer within an edge sequence is checked only if its hash value exists in the stored hash values. This check can be done in $O(mk)$ time for all k-mers in all edges. Empirically, more than 99.99% of k-mers that pass the rolling hash check are also selected k-mers. This does not affect the theoretical runtime of the algorithm but in practice it leads to a significant speedup.

Graph construction. The 128-bit hashes are used as the nodes of the graph. Edges are added whenever two hashes are adjacent to each other in a read. The constructed graph is then processed by condensing non-branching paths into *unitigs*. After this, unitigs are filtered based on a user given unitig abundance cutoff. Unitigs whose average coverage is less than the cutoff are discarded. In addition, edges whose coverage is less than the cutoff are also discarded. After unitig and edge removal the non-branching paths are again condensed into unitigs. The 128-bit hashes are transformed back to base pair sequences and homopolymer runs are decompressed. Finally, the graph is written in GFA format (Li, 2016).

Storing sequences. The base pair sequences of the selected k-mers are stored in memory as a store that contains a list of contiguous blocks. When a k-mer is added to the store, if the k-mer has overlap with the most recently added k-mer, the non-overlapping part is appended to the contiguous block. That is, the overlapping part is only stored once. If the k-mer does not overlap with the most recently added k-mer, a new block is started. The sequences are stored in homopolymer compressed format, with the lengths of the homopolymer runs stored separately. In practice this means that adjacent k-mers from the same read can be stored efficiently without duplicating the overlapping sequences, and moving from one read to another will almost certainly

start a new contiguous block.

Homopolymer run length consensus. When storing the sequences, homopolymer run lengths are also stored. Each stored base pair also has a sum of run lengths s stored in a 16-bit integer and a base pair count c stored in an 8-bit integer. When a k-mer is read from the input, for every base pair in the k-mer, the base pair count c of the associated base pair is incremented by one, and the homopolymer run length is added to the sum of run lengths s. If adding a k-mer would lead to an overflow of s or c, the run lengths for that k-mer are ignored and s and c are not updated. The run length consensus of each base pair is taken from the average $\frac{s}{c}$ rounded to the nearest integer. For example, if the input sequences are CAAAATTA and CAATTA, they would be stored as the base pair sequence CATA, sum of run lengths $s = [2, 6, 4, 2]$, and base pair counts $c = [2, 2, 2, 2]$ and their consensus would be CAAATTA. The run length consensus can optionally be disabled to reduce memory use, which is intended for the case when the input reads are already homopolymer compressed.

Runtime. Assuming no sequencing errors and given a genome size g, genomic coverage c, k-mer size k and window size w, the number of selected minimizers is m with $O(m) = O(\frac{g}{w})$ assuming the minimizer winnowing hash is random. The runtime of minimizer winnowing is $O(gc)$. Hashing the selected k-mers is $O(kcm) = O(\frac{kcg}{w})$. Cleaning transitive edges requires $O(km) = O(\frac{kg}{w})$ for the selected minimizers, and $O(kmk) = O(\frac{k^2g}{w})$ for k-mers which share their rolling hash value with a selected minimizer. Graph construction is $O(m) = O(\frac{g}{w})$. In total the runtime is $O(\frac{k^2g}{w} + \frac{kcg}{w} + gc)$. In practice the $\frac{k^2g}{w}$ term has a tiny constant factor and the runtime is dominated by the $O(\frac{kcg}{w})$ term.

Assuming no sequencing errors and a constant read length $r > k + w$, the memory use of MBG is $O(g + \frac{mk}{r-k-w} + m) = O(g + \frac{gk}{w(r-k-w)} + \frac{g}{w})$. In practice increasing w reduces memory use significantly.

B. Experimental setup

We used MBG version 1.0.1 from Bioconda. We used BCalm2 version 2.2.3 compiled with the option DKSIZE_LIST="32 64 96 128 160 192 224 256 320 512 1024 2048 3072 4096" to support higher k-mer sizes. All experiments were ran on a computing server with 48 Intel(R) Xeon(R) E7-8857 v2 CPUs and 1.5Tb of RAM. BCalm2 was given one thread in the command line invocation, and MBG is single threaded. Runtime and memory use was measured with "/usr/bin/time -v" in all experiments.

Comparison to existing tools. We compared MBG to BCalm2 (Chikhi *et al.*, 2016) for building graphs. Table 1 contains the results. We used HiFi data from *E. coli*[1], containing 290x coverage HiFi reads. We randomly downsampled the reads to 29x

[1]SRA accession number SRR10971019

coverage. We varied the window size parameter w for MBG from 1, resulting in an edge-centric de Bruijn graph, to higher values resulting in graphs of various sparsity. The k-mer abundance threshold was set to 3 for BCalm2, and the unitig average abundance threshold was set to 3 for MBG.

Due to the average density of random minimizers of $w/2$, and the homopolymer compression reducing the average length of sequence by 1/4, the results for a de Bruijn graph with k-mer size k_{DBG} are most closely comparable to a sparse de Bruijn graph with $k_{MBG} = \frac{3}{4}k_{DBG} - \frac{w}{2}$. We tried different values of k_{MBG} and w which result in similar graph quality as predicted by the above equation, and which match the k_{DBG} given to BCalm2. We also tried using equal values of $k = 61$, 91 and 127 for both MBG and BCalm2 while varying w for MBG. Finally, we tried higher k values up to 3001 for BCalm and up to 3501 for MBG.

Since the N50 of the $k = 2001$ and $k = 2501$ graphs matches the *E. coli* genome size, we evaluated their correctness by running QUAST (Gurevich *et al.*, 2013) on the *E. coli* K-12 substring MG1665 reference genome[2], and a de novo HiCanu (Nurk *et al.*, 2020) assembly of the same HiFi reads. The results were the same for $k = 2001$ and $k = 2501$ graphs produced by MBG. When compared to the reference genome, QUAST reported 8 misassemblies for both the MBG contigs and the HiCanu de novo assembly, all at the same locations. On the other hand the MBG contigs and the HiCanu de novo assembly were structurally consistent with each others. We suspect that the difference is due to the sequenced strain having differences to the strain used for constructing the reference genome.

Assembly error rates. We ran QUAST (Gurevich *et al.*, 2013) on all of the *E. coli* assemblies to evaluate the error rates of the assembled contigs using the *E. coli* K-12 substring MG1665 reference genome[3]. Most errors are expected to be wrong homopolymer run lengths. These are usually reported as indels, however the case where two adjacent runs have an extra character on one run and a missing character on the other may be reported as a mismatch. To include this case as well, we evaluated the error rates in two settings: first, using the sequences as is, and second, by homopolymer compressing both the assembled contigs and the reference and evaluating using the homopolymer compressed sequences. The difference in error rates between the two settings shows how many errors are caused by incorrect homopolymer run lengths, while the homopolymer compressed case measures errors unrelated to homopolymer runs. We take the error rate difference between the two settings as the homopolymer run length error rate, and the error rate of the homopolymer compressed setting as the error rate of all other errors.

Table 2 shows the results. Increasing w while keeping k constant degrades the homopolymer run length consensus accuracy, resulting in an increase in the homopolymer run length error rate but no effect on other types of errors. Taking the $k = 2000, w = 2000$ as a representative case of typical parameters, we estimate that homopolymer errors account for 99.6% of all errors, with a total error rate of $4.96 * 10^{-4}$ consisting

[2]GenBank accession U00096.2
[3]GenBank accession U00096.2

of a homopolymer run length error rate of $4.95 * 10^{-4}$ and an error rate of $1.8 * 10^{-6}$ for all other errors, corresponding to quality values of QV=33 in the default setting and QV=57 in the homopolymer compressed setting. In the homopolymer compressed setting, the $k = 2000, w = 2000$ assembly had just 3 substitutions and 3 indel errors over the entire *E. coli* genome, for a total error rate of $1.8 * 10^{-6}$. For comparison, a de novo HiCanu (Nurk *et al.*, 2020) (version 2.1.1) assembly of the same reads with the default HiFi assembly parameters has an error rate of $2.9 * 10^{-5}$ in the default setting and $5.2 * 10^{-6}$ in the compressed setting.

Whole human genome HiFi. We ran MBG on whole human genome HiFi data from the individual HG002. We used HiFi reads from the Human Pangenome Reference Consortium HG002 data freeze v1.0 (Wenger *et al.*, 2019)[4]. The reads contain 50x coverage HiFi reads with sizes ranging from 15kbp to 25kbp. For comparison we also ran BCalm2 (Chikhi *et al.*, 2016) on the same reads with $k = 127$. We did not run BCalm2 with higher k since the results of the *E. coli* experiment suggest the runtime would be prohibitive.

[4]Libraries m64012_190920_173625, m64012_190921_234837, m64015_190920_185703, m64015_190922_010918, m64011_190712_225711, m64011_190726_220327

Dataset	Tool	k	w	CPU-time	Memory (Gb)	N50
E. coli	BCalm2	61	-	0:00:59	1.1	1 025
		91	-	0:01:18	1.6	1 080
		127	-	0:01:40	2.0	1 212
		501	-	0:17:11	3.6	4 999
		1001	-	1:42:26	3.5	13 688
		2001	-	8:12:45	3.8	5 908
		3001	-	10:33:11	4.0	4 393
E. coli	MBG	61	1	0:01:33	3.2	73 728
		61	10	0:00:25	0.6	82 427
		61	20	0:00:16	0.3	82 418
		61	30	0:00:13	0.3	82 394
		91	1	0:01:46	3.5	117 742
		91	10	0:00:28	0.7	117 724
		91	20	0:00:18	0.4	117 742
		91	30	0:00:14	0.3	125 699
		127	1	0:01:53	3.9	132 765
		127	10	0:00:30	0.8	132 569
		127	20	0:00:17	0.4	132 766
		128	30	0:00:13	0.3	132 764
		45	1	0:01:46	2.8	60 479
		41	10	0:00:32	0.5	59 657
		35	20	0:00:20	0.3	57 134
		31	30	0:00:18	0.2	34 101
		69	1	0:01:44	3.1	82 820
		65	10	0:00:34	0.6	82 810
		59	20	0:00:23	0.4	73 682
		55	30	0:00:20	0.3	78 679
		95	1	0:02:13	3.4	117 784
		85	20	0:00:25	0.4	125 638
		81	30	0:00:19	0.3	117 643
E. coli	MBG	501	500	0:00:10	0.12	177 653
		1001	1000	0:00:09	0.13	698 111
		1501	1500	0:00:09	0.14	1 517 634
		2001	2000	0:00:08	0.14	4 639 237
		2501	2500	0:00:08	0.14	4 644 046
		3001	3000	0:00:08	0.14	4 090 727
		3501	3500	0:00:07	0.14	392 371
HG002	BCalm2	127	-	32:00:32	6.4	249
HG002	MBG	501	500	4:10:28	123.7	2 012
		1001	1000	4:38:40	132.6	4 501
		2001	2000	4:07:22	138.5	12 095
		3001	3000	3:57:59	137.3	23 104
		4001	4000	3:35:03	120.4	26 736
		5001	5000	2:06:52	68.9	20 699
		501	100	6:50:21	269.0	1 784
		1001	200	6:16:38	244.2	3 749
		2001	400	6:01:18	250.0	8 669
		3001	600	6:05:17	243.9	15 085
		4001	800	3:55:50	245.3	23 868
		5001	1000	4:47:44	244.1	33 649

Table 1: Experimental results

		Default			Compressed		
k	w	Subst.	Indel	Total	Subst.	Indel	Total
61	1	0.31	3.37	3.68	0	0.06	0.06
61	10	0.62	11.87	12.49	0	0.06	0.06
61	20	0.59	15.35	15.94	0	0.06	0.06
61	30	0.53	17.57	18.1	0	0.06	0.06
91	1	0.29	11.54	11.83	0	0.06	0.06
91	10	0.5	14.3	14.8	0	0.06	0.06
91	20	0.61	15.27	15.88	0	0.06	0.06
91	30	0.61	17.27	17.88	0	0.06	0.06
127	1	0.39	87.36	87.75	0	0.06	0.06
127	10	0.44	12.97	13.41	0	0.06	0.06
127	20	0.46	12.47	12.93	0	0.06	0.06
127	30	0.35	13.33	13.68	0	0.06	0.06
45	1	0.29	3.44	3.73	0	0.06	0.06
41	10	0.71	14.72	15.43	0	0.06	0.06
35	20	0.4	16.12	16.52	0	0.06	0.06
31	30	0.84	18.12	18.96	0	0.03	0.03
69	1	0.26	11.74	12	0	0.06	0.06
65	10	0.37	10.45	10.82	0	0.06	0.06
59	20	0.73	16.61	17.34	0	0.06	0.06
55	30	0.64	19.79	20.43	0	0.06	0.06
95	1	0.29	11.1	11.39	0	0.06	0.06
85	20	0.55	15.96	16.51	0	0.06	0.06
81	30	0.35	16.43	16.78	0	0.06	0.06
501	500	0.97	65.94	66.91	0.21	0.15	0.36
1001	1000	1.16	65.95	67.11	0.15	0.09	0.24
1501	1500	0.88	59.58	60.46	0.15	0.09	0.24
2001	2000	0.78	48.86	49.64	0.09	0.09	0.18
2501	2500	0.67	43.97	44.64	0.09	0.09	0.18
3001	3000	0.52	32.69	33.21	0.09	0.09	0.18
3501	3500	0.5	35.3	35.8	0.15	0.15	0.3

Table 2: Error rates of the *E. coli* assemblies measured by substitution errors per 100kbp (Subst.), indel errors per 100kbp (Indel) and total error rate per 100kbp (Total), separated into the setting where the reference and contigs are not homopolymer compressed (Default) and the setting where the reference and contigs are homopolymer compressed (Compressed).

References

Chikhi, R., Limasset, A., and Medvedev, P. (2016). Compacting de Bruijn graphs from sequencing data quickly and in low memory. *Bioinformatics*, **32**(12), i201–i208.

Gurevich, A., Saveliev, V., Vyahhi, N., and Tesler, G. (2013). QUAST: quality assessment tool for genome assemblies. *Bioinformatics*, **29**(8), 1072–1075.

Li, H. (2016). Minimap and miniasm: fast mapping and de novo assembly for noisy long sequences. *Bioinformatics*, **32**(14), 2103–2110.

Mohamadi, H., Chu, J., Vandervalk, B. P., and Birol, I. (2016). ntHash: recursive nucleotide hashing. *Bioinformatics*, **32**(22), 3492–3494.

Nurk, S., Walenz, B. P., Rhie, A., Vollger, M. R., Logsdon, G. A., Grothe, R., Miga, K. H., Eichler, E. E., Phillippy, A. M., and Koren, S. (2020). HiCanu: accurate assembly of segmental duplications, satellites, and allelic variants from high-fidelity long reads. *BioRxiv*.

Schleimer, S., Wilkerson, D. S., and Aiken, A. (2003). Winnowing: local algorithms for document fingerprinting. In *Proceedings of the 2003 ACM SIGMOD international conference on Management of data*, pages 76–85.

Wenger, A. M., Peluso, P., Rowell, W. J., Chang, P.-C., Hall, R. J., Concepcion, G. T., Ebler, J., Fungtammasan, A., Kolesnikov, A., Olson, N. D., *et al.* (2019). Accurate circular consensus long-read sequencing improves variant detection and assembly of a human genome. *Nature biotechnology*, **37**(10), 1155–1162.
